# Protein density, carbohydrate quality, and frailty-state transitions among adults aged 50 years or older: a continuous-time multistate analysis of the health and retirement study

**DOI:** 10.3389/fnut.2026.1926576

**Published:** 2026-09-24

**Authors:** Jiao Wang

**Affiliations:** Heping Hospital Affiliated to Changzhi Medical College, Changzhi, China

**Keywords:** carbohydrate quality, continuous-time multistate Markov model, frailty, health and retirement study, nutrition, protein density

## Abstract

**Background:**

Frailty is a dynamic condition in which individuals may deteriorate, recover, remain stable, or die. Although healthier dietary patterns have been associated with a lower risk of frailty, less is known about their associations with specific frailty-state transitions. We examined whether a study-specific score emphasizing protein density and carbohydrate quality was associated with transitions between frailty states.

**Methods:**

We linked the 2013 Health and Retirement Study nutrition assessment to repeated frailty assessments from 2014 to 2021 among participants aged 50 years or older. The study-specific score combined higher densities of protein, fiber, and whole-grain densities with lower densities of added-sugar and glycemic-load densities. Frailty was assessed using a published 28-item deficit-accumulation index. The primary continuous-time multistate model allowed baseline transition intensities to change at 2 and 4 years and adjusted for age, sex, education, body mass index (BMI), ethnicity, Hispanic ethnicity, smoking, alcohol consumption, and moderate and light physical activity. Dietary components were also examined individually and jointly.

**Results:**

The primary analysis included 6,982 participants aged 50–102 years (mean, 69.0 years; 60.3% aged 65 years or older), with 25,108 person-state records and 1,487 deaths. Including the score improved model fit (likelihood-ratio [LR], 24.08; degrees of freedom [df], 7; *p* = 0.001). Each 1-standard deviation (SD) higher score was associated with lower robust-to-pre-frail intensity (hazard ratio [HR], 0.933; nominal 95% confidence interval [CI], 0.875–0.995) and pre-frail-to-death intensity (HR, 0.809; 95% CI, 0.662–0.989). Confidence intervals included 1 for the remaining worsening, recovery, and death transitions. No individual component association remained statistically significant after mutual adjustment and false-discovery-rate correction.

**Conclusion:**

After comprehensive adjustment, a higher study-specific score was associated with lower robust-to-pre-frail and pre-frail-to-death intensities, but evidence for other transitions was inconclusive. The score remains unvalidated, and these findings do not establish a causal effect of diet on frailty transitions.

## Introduction

Frailty reflects diminished physiologic reserve and heightened vulnerability to stressors beyond that attributable to chronological age, individual diseases, or disability alone (1, 2). Two complementary conceptual approaches have shaped the field. The physical phenotype emphasizes weakness, slowness, exhaustion, low activity, and weight loss, whereas deficit-accumulation indices quantify health deficits across multiple systems (3, 4). In longitudinal population studies, the deficit-accumulation approach provides a graded measure of vulnerability that can be repeatedly assessed over time.

Frailty is clinically and epidemiologically important not only because it predicts adverse outcomes, but also because its severity may change over time. Although greater frailty burden predicts mortality and other adverse outcomes across cohorts and meta-analyses, frailty does not invariably progress: older adults may deteriorate, recover, remain stable, or die between assessments (5–10). A transition-based perspective is particularly relevant to nutritional epidemiology because associations with deterioration, recovery, and death may differ across these distinct outcomes (9, 11, 12).

Diet is a modifiable factor that may shape frailty trajectories. Diet quality is inherently multidimensional. Established indices, including the Mediterranean, dietary approaches to stop hypertension (DASH), and Healthy Eating Index measures, integrate multiple foods, nutrients, and adherence targets rather than relying on a single dietary component. Prospective studies and meta-analyses have linked Mediterranean-style and other healthier dietary patterns, protein intake, fruit and vegetable consumption, and nutrient adequacy to frailty-related outcomes (13–20). However, most studies have examined prevalent or incident frailty at subsequent assessment points, making it difficult to determine whether diet is associated with specific transitions, such as slower deterioration, greater recovery, or lower mortality.

We therefore focused on a targeted dietary construct rather than overall diet quality. Protein density may support muscle maintenance, whereas fiber, whole grains, added sugars, and glycemic load capture complementary aspects of carbohydrate quality and glycemic burden (21–29). A composite score can be used to assess whether this specific dietary profile is associated with frailty dynamics, but it is not equivalent to a validated Mediterranean, DASH, or Healthy Eating Index score.

We used the Health and Retirement Study (HRS), a longitudinal study of aging in the United States. We examined whether a study-specific protein-density and carbohydrate-quality score was associated with frailty-state transitions over up to 7.1 years (30, 31). Frailty states were defined using a 28-item deficit-accumulation frailty index constructed according to published HRS methods (32). We hypothesized that higher scores would be associated with lower transition intensities for frailty worsening and death and higher transition intensities for recovery.

## Methods

### Study design and analytic sample

This secondary longitudinal cohort analysis used data from the HRS. We linked the 2013 nutrition assessment to frailty assessments at waves 12–15, corresponding nominally to 2014, 2016, 2018, and 2020. Actual interview dates ranged from March 2014 to May 2021. Because HRS may include spouses or partners younger than the age-eligible respondents, eligibility was restricted to participants aged 50 years or older at wave 12 (30, 31).

Of 8,035 nutrition records, 8,021 were successfully linked to RAND HRS data. We excluded 445 participants with missing baseline age and 142 participants aged younger than 50 years, leaving 7,434 age-eligible participants. We then sequentially excluded 3 participants without a valid wave 12 frailty state, 124 without complete core covariate data, and 246 with fewer than two valid frailty-state observations. An additional 79 participants with missing values for covariates in the fully adjusted model were excluded. The primary complete-case sample comprised 6,982 participants (Figure 1).

**Figure 1:**
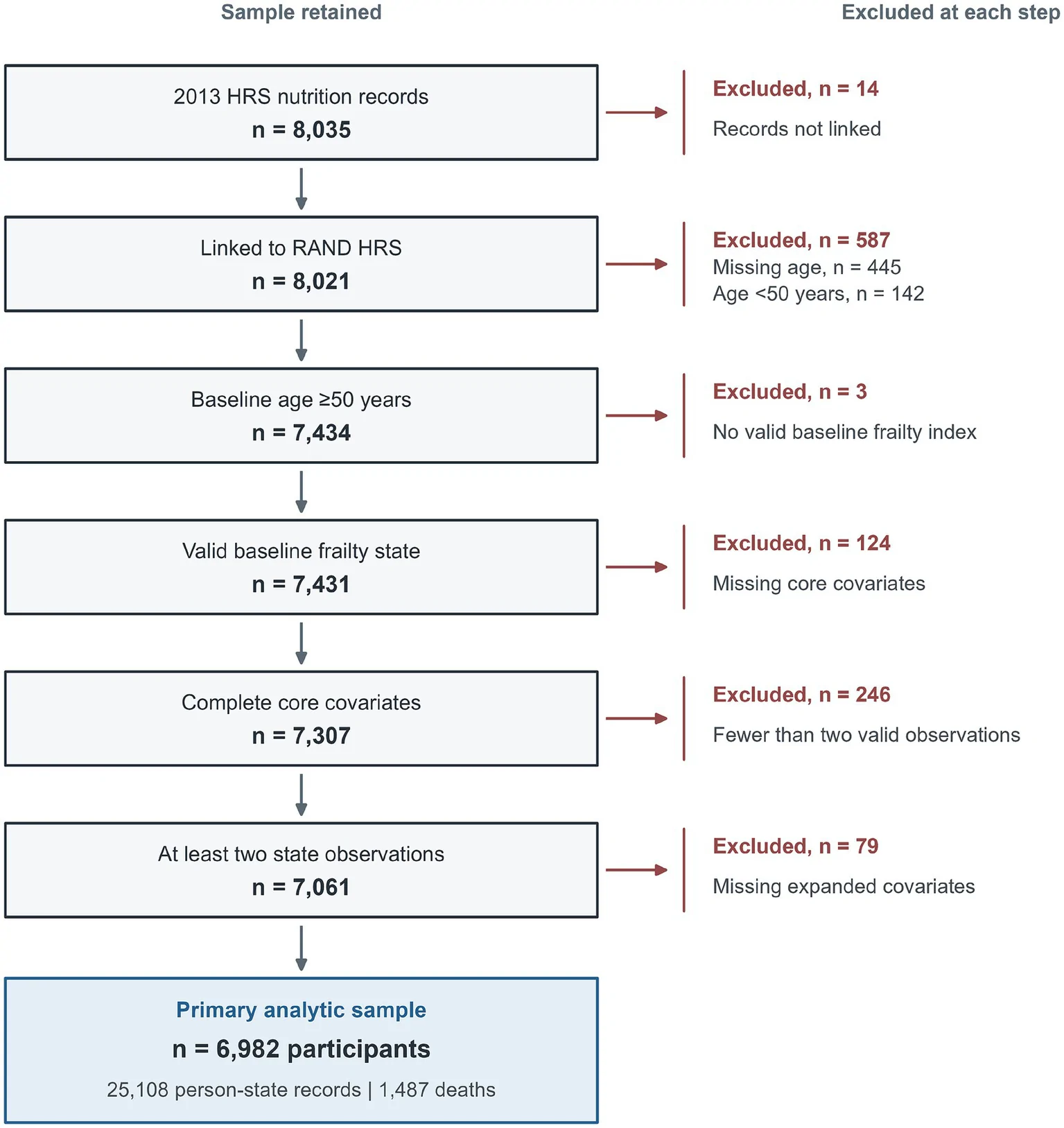
Sequential participant selection for the fully adjusted primary analysis. FI, frailty index; HRS, Health and Retirement Study.

The primary dataset contained 25,108 person-state records and 1,487 deaths. Participants’ baseline ages ranged from 50 to 102 years; 4,213 participants (60.3%) were aged 65 years or older. The maximum follow-up was 7.13 years. Death was modeled as an absorbing state.

### Dietary exposure

The primary exposure was a study-specific, hypothesis-driven score of protein density and carbohydrate quality, derived from the 2013 HRS nutrition assessment. This score represents a targeted dietary profile rather than a validated instrument or a comprehensive measure of overall diet quality. The five components were selected from prior evidence linking protein adequacy and carbohydrate quality to physical and metabolic reserve (21–29).

Protein, fiber, whole-grain, added-sugar, and glycemic-load variables were expressed per 1,000 kcal. Each component was standardized across all 8,035 nutrition records. The equally weighted score was calculated as the mean of the z scores for protein, fiber, and whole-grain intake and the reverse-coded z scores for added sugar intake and glycemic load. All five components were complete. Component weights were independent of frailty outcomes. The composite was standardized using the mean and SD among the 7,061 participants eligible for core-adjusted analyses; one SD corresponded to 0.616 score units. This scale was used in all model comparisons. Quartiles and prediction percentiles were calculated among the 6,982 participants in the primary sample (Supplementary material (Methods); Supplementary Table S1).

### Outcome definition: frailty index and frailty states

Frailty was assessed using a 28-item deficit-accumulation frailty index constructed from HRS variables according to published methods (32). The same multidomain deficits were available consistently across the four waves, whereas complete measures required to construct Fried phenotype performance were unavailable for every participant at every wave. The index covered chronic disease, memory-related disease, sensory impairment, general health, activities of daily living, mobility, depressive symptoms, and cognition. Because it includes ADL and IADL limitations, the index captures multidomain vulnerability that partially overlaps with disability. Supplementary Table S2 provides detailed coding definitions.

At each wave, the frailty index was calculated as the sum of observed deficits divided by the number of observed items. Calculation required at least 22 of 28 observed items, thereby allowing up to six missing items; otherwise, the index was set to missing. Supplementary Table S3 reports wave-specific availability and distributions.

Following the published HRS frailty-index implementation, we defined three living frailty states (32). Participants were classified as robust for values below 0.10, pre-frail for values from 0.10 to below 0.25, and frail for values of 0.25 or higher. Death constituted the fourth, absorbing state. Although categorization results in some loss of continuous information, it defines a finite and clinically interpretable state space for deterioration, recovery, and death.

### Continuous-time multistate Markov model

We fitted continuous-time multistate Markov models using the msm package in R. The allowed transitions were robust to pre-frail, pre-frail to robust, pre-frail to frail, frail to pre-frail, and transitions from each living state to death.

Direct transitions between robust and frail states were not parameterized. Accordingly, observed non-adjacent changes between biennial visits were treated as interval changes that could have occurred through the unobserved intermediate state.

Follow-up was measured in years from each participant’s wave-12 interview. Interview dates were obtained from RAND monthly midpoint variables. Deaths were modeled as exact events using the recorded RAND death dates. The primary model allowed baseline transition intensities to vary across three follow-up periods: 0 to <2, 2 to <4, and at least 4 years. Dietary associations were estimated separately for each transition and assumed to remain constant across the three follow-up periods.

The primary model adjusted for age, sex, education, baseline BMI, ethnicity, Hispanic ethnicity, current smoking, alcohol consumption, and moderate and light physical activity. These covariates were included as potential demographic, socioeconomic, and behavioral confounders, with separate coefficients for each allowed transition. Physical-activity frequencies were modeled as standardized ordinal variables. Core-adjusted models included age, sex, education, and baseline BMI. Core-adjusted and time-homogeneous models were fitted to the same complete-case sample for comparison. A sensitivity analysis omitted BMI because it may mediate long-term dietary associations. Models were conditional on the initial frailty state; continuous baseline FI was not included as an additional covariate.

We estimated state-occupation probabilities at 6 years for the 10th and 90th percentiles of the dietary profile score. Other covariates were fixed at their participant-level sample means, including mean indicators for categorical variables. Piecewise-model probabilities were calculated from the time-ordered product of transition matrices across the three periods. These predictions describe a reference covariate profile; they are neither population-standardized estimates nor estimates of causal effects. Supplementary Tables S4, S5 describe participant selection, ascertainment of study dates, and model diagnostics.

### Model checking and sensitivity analyses

Likelihood-ratio tests compared nested models fitted to the same participants and observations. We compared piecewise and time-homogeneous models under full adjustment, and assessed models with continuous or quartile dietary scores against the corresponding model without the dietary score. Comparisons of Akaike information criterion (AIC) values also used the same sample. Transition-specific 95% CIs for the composite score were nominal and were not adjusted for multiplicity. Numerical convergence was assessed using optimizer status, Hessian and gradient diagnostics, and finite confidence intervals.

Sensitivity analyses used time-homogeneous or core-adjusted models, required at least 25 of 28 observed FI items, omitted BMI, or applied delayed entry from year 2. For delayed entry, follow-up began at the first available living-state assessment at or after year 2, with at least one subsequent state observation required. We also examined each dietary component separately and all five components jointly, using the primary model’s time periods and covariate adjustment. Component HRs were expressed per 1-SD higher intake density in the primary sample; added sugar and glycemic load were not reverse-coded. Benjamini-Hochberg correction was applied to 35 component-transition tests within each exploratory family: the separate-component models and the mutually adjusted model.

Data preparation and analysis used R and Python. Continuous-time models were fitted in R version 4.6.0 using msm version 1.8.2. Hazard ratios for transition intensities are reported with 95% confidence intervals and interpreted as associations rather than causal effects.

## Results

### Analytic sample and baseline characteristics

The primary sample included 6,982 participants, 25,108 person-state records, and 1,487 deaths. Mean baseline age was 69.00 years (SD, 10.49); 58.8% were female and 60.3% were aged 65 years or older. At baseline, 2,196 participants were robust (31.5%), 2,664 were pre-frail (38.2%), and 2,122 were frail (30.4%; Figure 1).

Baseline characteristics differed across dietary profile score quartiles. Mean FI was 0.17 in Q4 and 0.23 in Q1. Table 1 summarizes baseline characteristics; Supplementary Table S6 provides detailed demographic and lifestyle characteristics, including all covariates in the primary model.

**Table 1:** Baseline characteristics by quartile of dietary profile score.

Characteristic	Overall	Q1	Q2	Q3	Q4	*p*
Participants	6,982	1,746	1,745	1,745	1,746	
Dietary profile score	0.00 (0.62)	−0.80 (0.42)	−0.14 (0.11)	0.22 (0.10)	0.73 (0.28)	<0.001
Age, years	69.00 (10.49)	68.89 (10.84)	69.83 (10.61)	69.40 (10.30)	67.89 (10.10)	<0.001
Female	4,103 (58.8%)	937 (53.7%)	1,010 (57.9%)	1,060 (60.7%)	1,096 (62.8%)	<0.001
Education, years	12.89 (3.03)	12.30 (2.78)	12.83 (2.88)	13.26 (2.91)	13.19 (3.42)	<0.001
BMI, kg/m2	28.60 (6.08)	28.44 (6.30)	28.58 (5.96)	28.76 (6.01)	28.60 (6.05)	0.493
Current smoking	771 (11.0%)	324 (18.6%)	209 (12.0%)	127 (7.3%)	111 (6.4%)	<0.001
Frailty index	0.20 (0.16)	0.23 (0.18)	0.21 (0.16)	0.19 (0.15)	0.17 (0.15)	<0.001
Robust	2,196 (31.5%)	460 (26.3%)	506 (29.0%)	566 (32.4%)	664 (38.0%)	<0.001
Pre-frail	2,664 (38.2%)	624 (35.7%)	675 (38.7%)	693 (39.7%)	672 (38.5%)	
Frail	2,122 (30.4%)	662 (37.9%)	564 (32.3%)	486 (27.9%)	410 (23.5%)	
Death during follow-up	1,487 (21.3%)	472 (27.0%)	411 (23.6%)	349 (20.0%)	255 (14.6%)	<0.001

### Observed frailty-state transitions

Both deterioration and recovery were observed between successive assessments. Table 2 presents the observed state changes, including unchanged states and deaths, in the primary sample.

**Table 2:** Observed frailty-state transitions between HRS visits.

From	To	*n*	Row %	Model representation
robust	robust	4,002	73.7	
robust	pre-frail	1,213	22.3	
robust	frail	90	1.7	Latent intermediate state
robust	death	126	2.3	
pre-frail	robust	858	12.3	
pre-frail	pre-frail	4,473	63.9	
pre-frail	frail	1,302	18.6	
pre-frail	death	371	5.3	
frail	robust	20	0.4	Latent intermediate state
frail	pre-frail	806	14.2	
frail	frail	3,875	68.1	
frail	death	990	17.4	

The model represented non-adjacent living-state changes as passage through the unobserved intermediate state between visits (Table 2; Supplementary Table S7).

### Dietary profile score in the primary piecewise multistate model

In the primary piecewise model, each 1-SD higher dietary profile score was associated with lower robust-to-pre-frail intensity (HR, 0.933; 95% CI, 0.875–0.995). Higher scores were also associated with lower pre-frail-to-death intensity (HR, 0.809; 95% CI, 0.662–0.989; Figure 2; Table 3).

**Figure 2:**
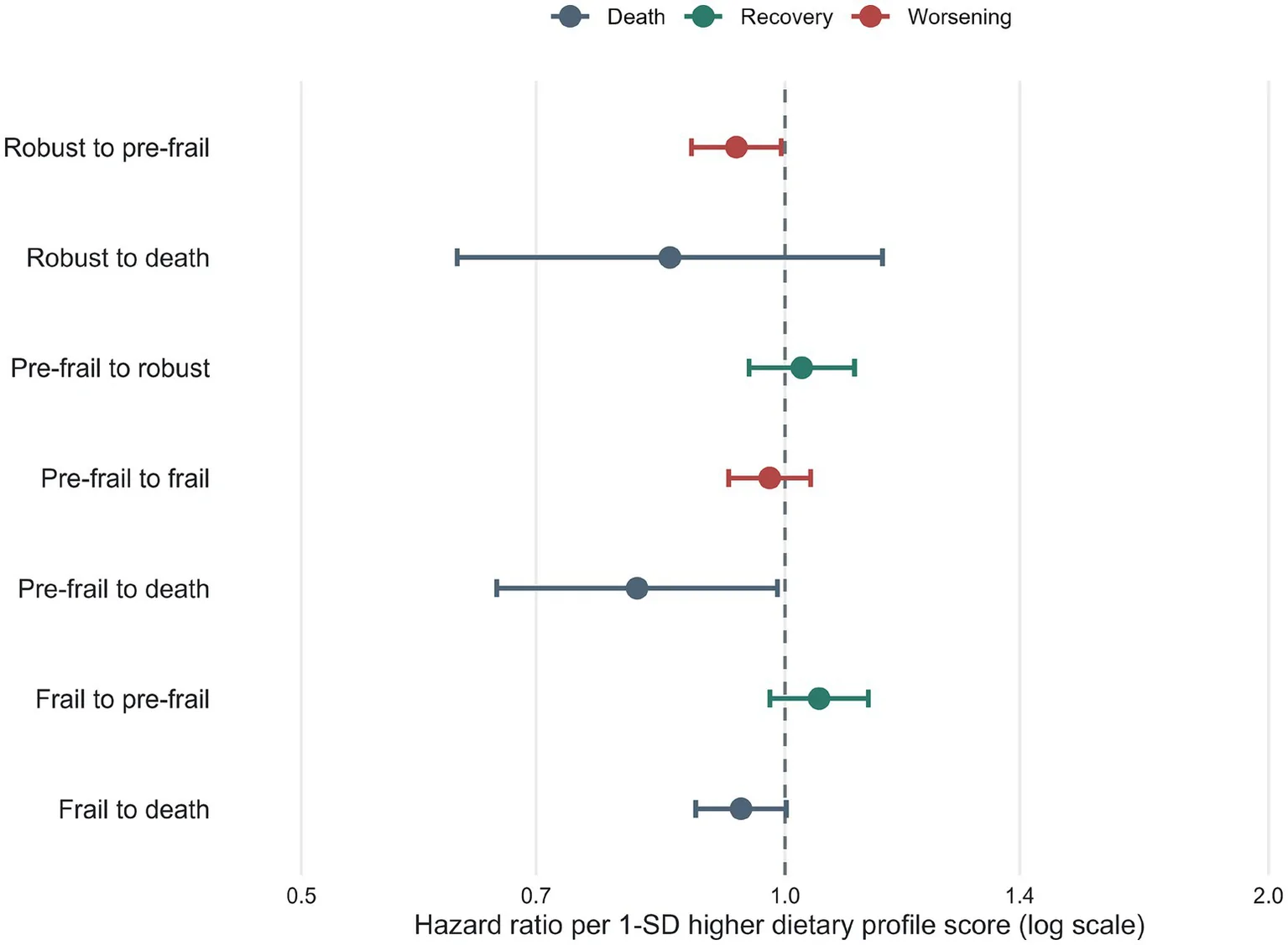
Transition-specific hazard ratios per 1-SD higher dietary profile score in the fully adjusted piecewise continuous-time multistate model. Points show HRs, and error bars show 95% confidence intervals.

**Table 3:** Primary fully adjusted piecewise model estimates per 1-SD higher dietary profile score.

Transition	HR	95% CI
Robust to pre-frail	0.933	0.875–0.995
Robust to death	0.848	0.625–1.150
Pre-frail to robust	1.024	0.950–1.105
Pre-frail to frail	0.978	0.922–1.038
Pre-frail to death	0.809	0.662–0.989
Frail to pre-frail	1.050	0.979–1.127
Frail to death	0.939	0.880–1.002

The nominal 95% CIs included 1 for pre-frail-to-frail progression, both recovery pathways, and transitions from the robust and frail states to death, indicating inconclusive evidence regarding these transitions.

### Predicted 6-year state probabilities

For an initially robust reference profile, the high-score scenario had a 6-year probability of robust status 6.7 percentage points higher, a probability of frail status 2.3 percentage points lower, and a probability of death 3.0 percentage points lower than the low-score scenario.

For an initially pre-frail reference profile, the high-score scenario had a probability of robust status 2.9 percentage points higher and a probability of death 3.7 percentage points lower. For an initially frail profile, the probability of pre-frail status was 3.4 percentage points higher and the probability of death was 4.3 percentage points lower. These are descriptive, model-based contrasts rather than intervention effects (Figure 3; Table 4).

**Figure 3:**
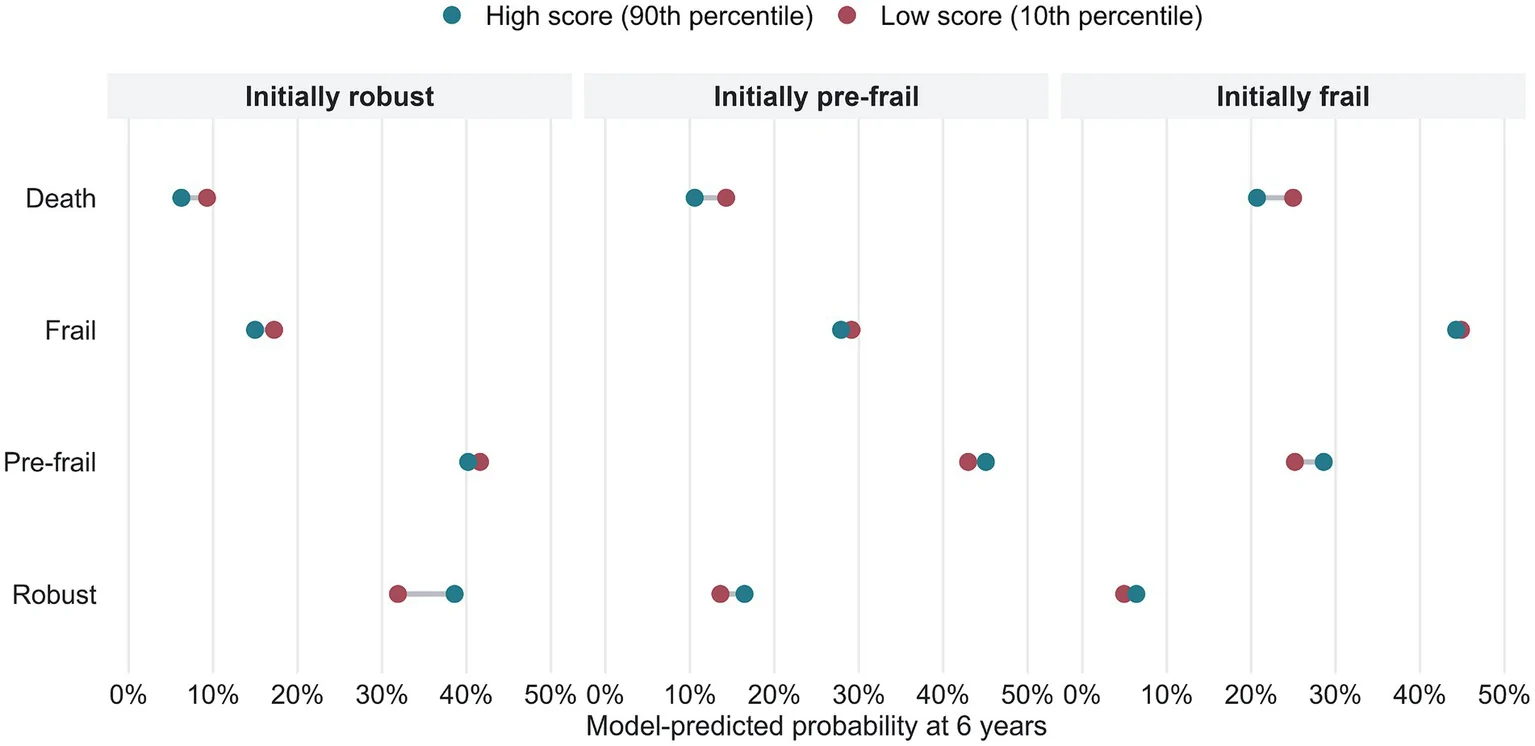
Six-year state-occupation probabilities from the fully adjusted piecewise model for low and high dietary scores (10th and 90th percentiles). Other covariates are fixed at their participant-level means. Connected points compare the two score profiles and do not denote confidence intervals.

**Table 4:** Six-year predicted state-occupation probabilities from the fully adjusted piecewise model.

Initial state	State at 6 years	Low score	High score	Difference, percentage points
robust	robust	31.9%	38.6%	+6.7
robust	pre-frail	41.6%	40.2%	−1.4
robust	frail	17.2%	15.0%	−2.3
robust	death	9.3%	6.2%	−3.0
pre-frail	robust	13.6%	16.5%	+2.9
pre-frail	pre-frail	43.0%	45.1%	+2.1
pre-frail	frail	29.1%	27.9%	−1.2
pre-frail	death	14.3%	10.6%	−3.7
frail	robust	5.0%	6.4%	+1.4
frail	pre-frail	25.2%	28.6%	+3.4
frail	frail	44.9%	44.3%	−0.6
frail	death	25.0%	20.7%	−4.3

### Dietary profile specification and model fit

Table 5 reports Q4 vs. Q1 estimates under the same fully adjusted piecewise specification. Using the same sample, the fully adjusted piecewise model fitted better than the time-homogeneous model (LR, 354.02; df, 14; *p* < 0.001). Corresponding AIC values were 31,763.53 and 32,089.56. Including the continuous score improved fit relative to the piecewise model without the dietary score (LR, 24.08; df, 7; *p* = 0.001). The quartile comparison yielded LR 32.31 (df, 21; *p* = 0.054). Table 6 and Supplementary Table S8 provide the model comparisons. Supplementary Table S9 reports estimates and confidence intervals for all quartiles.

**Table 5:** Highest vs. lowest quartile of the dietary profile score.

Transition (Q4 vs. Q1)	HR	95% CI	*p*
Robust to pre-frail	0.807	0.680–0.958	0.014
Robust to death	0.527	0.213–1.303	0.166
Pre-frail to robust	1.085	0.883–1.335	0.437
Pre-frail to frail	0.905	0.766–1.070	0.244
Pre-frail to death	0.693	0.401–1.197	0.189
Frail to pre-frail	1.142	0.926–1.408	0.214
Frail to death	0.844	0.693–1.028	0.091

**Table 6:** Global model comparison tests.

Comparison	LR	df	*p*
Continuous score vs. score omitted	24.08	7	0.001
Score quartiles vs. score omitted	32.31	21	0.054
Piecewise vs. time-homogeneous, full adjustment	354.02	14	<0.001

### Sensitivity and component-specific analyses

With stricter FI completeness, nominal CIs remained below 1 for the robust-to-pre-frail and pre-frail-to-death transitions. When BMI was omitted, CIs for these two associations included 1, whereas the frail-to-death CI excluded 1 (Table 7). Supplementary Table S9 also presents core-adjusted and time-homogeneous estimates for the same complete-case sample.

**Table 7:** Sensitivity analyses of transition-specific dietary profile associations.

Transition	Primary piecewise	Time-homogeneous	FI > = 25 items	Entry from year 2	BMI omitted
Robust to pre-frail	0.933 (0.875–0.995)	0.934 (0.876–0.996)	0.935 (0.876–0.997)	0.940 (0.859–1.028)	0.939 (0.880–1.000)
Robust to death	0.848 (0.625–1.150)	0.856 (0.617–1.187)	0.847 (0.629–1.141)	0.849 (0.563–1.281)	0.847 (0.616–1.164)
Pre-frail to robust	1.024 (0.950–1.105)	1.025 (0.950–1.105)	1.030 (0.954–1.111)	1.039 (0.940–1.148)	1.009 (0.935–1.088)
Pre-frail to frail	0.978 (0.922–1.038)	0.976 (0.920–1.035)	0.985 (0.928–1.045)	1.024 (0.945–1.110)	0.980 (0.924–1.040)
Pre-frail to death	0.809 (0.662–0.989)	0.831 (0.670–1.030)	0.798 (0.654–0.973)	0.787 (0.602–1.029)	0.827 (0.676–1.012)
Frail to pre-frail	1.050 (0.979–1.127)	1.049 (0.978–1.126)	1.055 (0.983–1.132)	1.059 (0.964–1.164)	1.039 (0.968–1.114)
Frail to death	0.939 (0.880–1.002)	0.944 (0.885–1.008)	0.942 (0.882–1.005)	0.939 (0.867–1.018)	0.920 (0.862–0.982)

Except for the homogeneous comparator, the models allow piecewise-constant baseline intensities and retain full adjustment unless explicitly stated.

The delayed-entry analysis included 5,920 participants and 932 deaths. Estimates were less precise, and all seven transition-specific CIs included 1. Overall, the associations were not consistently supported across sensitivity analyses.

In separate component models, only the association between higher fiber density and lower robust-to-pre-frail intensity remained statistically significant after false-discovery-rate correction (Supplementary Table S10). Joint inclusion of the five components improved model fit (LR, 96.94; df, 35; *p* < 0.001). However, no individual component association remained statistically significant after mutual adjustment and false-discovery-rate correction (Supplementary Table S11). Supplementary Table S12 presents period-specific baseline intensities, and Supplementary Table S13 reports component correlations and variance inflation factors.

## Discussion

Among adults aged 50 years or older, higher dietary profile scores were associated with lower robust-to-pre-frail and pre-frail-to-death intensities in the fully adjusted piecewise model. Evidence for the remaining progression, recovery, and death transitions was inconclusive. Several associations weakened with broader covariate adjustment, and the delayed-entry analysis did not provide consistent support for the primary findings.

This study extends prior work by treating frailty as a dynamic state process rather than a single endpoint. Previous studies have shown that frailty may improve or worsen and that these transitions carry prognostic information (9–11). By separating deterioration, recovery, and death, our analysis shows that the strength and robustness of dietary associations differ across transition pathways.

Prior studies have linked Mediterranean-style and other healthier dietary patterns to frailty risk (13–15, 18–20), and have examined protein intake and diet quality in relation to frailty outcomes (16, 17, 23). Our analysis examines a targeted dietary profile and distinguishes associations with deterioration, recovery, and death. Differences in exposure definitions, covariate adjustment, and frailty measurement limit direct comparisons with studies of incident frailty or established diet-quality indices.

The score represents a targeted dietary profile rather than a comprehensive measure of diet quality. Protein density relates to muscle maintenance (27–29), whereas the other components capture aspects of carbohydrate quality and glycemic burden (24, 25). Although joint inclusion of the components improved model fit, no individual component association remained statistically significant after mutual adjustment and false-discovery-rate correction. These findings do not establish which components account for the composite association, nor do they demonstrate synergy. External validation and comparison with established dietary indices remain necessary.

Deficit-accumulation indices and multistate models are well suited to repeated measures of aging. The frailty index provides a multidomain measure with transparent coding and missingness rules (3, 4). The continuous-time model estimates transition-specific intensities, accommodates irregular spacing between interviews, and treats death as an absorbing state (33–36). The modeled states therefore represent multidomain vulnerability rather than a purely physical frailty phenotype.

Strengths include repeated assessments in a large longitudinal cohort, a published 28-item frailty index, and explicit modeling of death as an absorbing state. Interview midpoint and recorded death dates defined follow-up intervals. The primary piecewise model accommodated time-varying baseline transition intensities.

Several limitations warrant consideration. The observational design precludes causal inference, and residual confounding remains possible. Baseline BMI and physical activity may reflect pre-existing frailty or consequences of earlier diet; the estimates are conditional associations rather than total causal effects. Diet was measured only once, in 2013, leaving dietary change, measurement error, and reverse causation unresolved. Delayed-entry analyses were conditional on survival and continued observation and do not exclude reverse causation. The equally weighted score is study-specific and unvalidated, and the component analyses do not establish its external validity. Only 60.3% of participants were aged 65 years or older, so the cohort represents middle-aged and older adults rather than an exclusively geriatric population. Muscle failure and sarcopenia were not evaluated. Because these conditions may precede or contribute to frailty, we could not determine whether dietary associations were independent of prior changes in muscle health (37). The FI includes ADL and IADL limitations and therefore partly overlaps with disability. Panel observations also require inference regarding unobserved transitions. The piecewise model relaxes time homogeneity but retains the Markov assumption and assumes constant transition-specific dietary HRs within each period. Complete-case selection and the absence of survey weighting may further limit generalizability.

In conclusion, higher dietary profile scores were associated with lower robust-to-pre-frail and pre-frail-to-death transition intensities, but these associations were not consistently supported across sensitivity analyses. Evidence for other transitions was inconclusive. Replication using validated, repeated dietary measures and direct assessments of muscle health is needed before considering implications for dietary interventions.

## Data Availability

The original contributions presented in the study are included in the article/Supplementary material, further inquiries can be directed to the corresponding author.

## References

[1] Clegg A Young J Iliffe S Rikkert MO Rockwood K. Frailty in elderly people. Lancet. (2013) 381:752–62. doi: 10.1016/S0140-6736(12)62167-9, 23395245 PMC4098658

[2] Fried LP Tangen CM Walston J Newman AB Hirsch C Gottdiener J . Frailty in older adults: evidence for a phenotype. J Gerontol A Biol Sci Med Sci. (2001) 56:M146–56. doi: 10.1093/gerona/56.3.m146, 11253156

[3] Rockwood K Mitnitski A. Frailty in relation to the accumulation of deficits. J Gerontol A Biol Sci Med Sci. (2007) 62:722–7. doi: 10.1093/gerona/62.7.722, 17634318

[4] Searle SD Mitnitski A Gahbauer EA Gill TM Rockwood K. A standard procedure for creating a frailty index. BMC Geriatr. (2008) 8:24. doi: 10.1186/1471-2318-8-24, 18826625 PMC2573877

[5] Song X Mitnitski A Rockwood K. Prevalence and 10-year outcomes of frailty in older adults in relation to deficit accumulation. J Am Geriatr Soc. (2010) 58:681–7. doi: 10.1111/j.1532-5415.2010.02764.x, 20345864

[6] Kojima G Iliffe S Walters K. Frailty index as a predictor of mortality: a systematic review and meta-analysis. Age Ageing. (2017) 47:193–200. doi: 10.1093/ageing/afx162 29040347

[7] Vermeiren S Vella-Azzopardi R Beckwée D Habbig A-K Scafoglieri A Jansen B . Frailty and the prediction of negative health outcomes: a meta-analysis. J Am Med Dir Assoc. (2016) 17:1163.e1–1163.e17. doi: 10.1016/j.jamda.2016.09.010 27886869

[8] Hoogendijk EO Afilalo J Ensrud KE Kowal P Onder G Fried LP. Frailty: implications for clinical practice and public health. Lancet. (2019) 394:1365–75. doi: 10.1016/S0140-6736(19)31786-6, 31609228

[9] Gill TM Gahbauer EA Allore HG Han L. Transitions between frailty states among community-living older persons. Arch Intern Med. (2006) 166:418–23. doi: 10.1001/archinte.166.4.418, 16505261

[10] Xue Q-L. The frailty syndrome: definition and natural history. Clin Geriatr Med. (2011) 27:1–15. doi: 10.1016/j.cger.2010.08.009, 21093718 PMC3028599

[11] Cesari M Gambassi G van Kan GA Vellas B. The frailty phenotype and the frailty index: different instruments for different purposes. Age Ageing. (2013) 43:10–2. doi: 10.1093/ageing/aft160, 24132852

[12] Hanlon P Nicholl BI Jani BD Lee D McQueenie R Mair FS. Frailty and pre-frailty in middle-aged and older adults and its association with multimorbidity and mortality: a prospective analysis of 493 737 UK biobank participants. Lancet Public Health. (2018) 3:e323–32. doi: 10.1016/S2468-2667(18)30091-4, 29908859 PMC6028743

[13] Talegawkar SA Bandinelli S Bandeen-Roche K Chen P Milaneschi Y Tanaka T . A higher adherence to a Mediterranean-style diet is inversely associated with the development of frailty in community-dwelling elderly men and women. J Nutr. (2012) 142:2161–6. doi: 10.3945/jn.112.165498, 23096005 PMC3497964

[14] León-Muñoz LM García-Esquinas E López-García E Banegas JR Rodríguez-Artalejo F. Major dietary patterns and risk of frailty in older adults: a prospective cohort study. BMC Med. (2015) 13:11. doi: 10.1186/s12916-014-0255-6, 25601152 PMC4298966

[15] Kojima G Avgerinou C Iliffe S Walters K. Adherence to Mediterranean diet reduces incident frailty risk: systematic review and Meta-analysis. J Am Geriatr Soc. (2018) 66:783–8. doi: 10.1111/jgs.15251, 29322507

[16] Beasley JM LaCroix AZ Neuhouser ML Huang Y Tinker L Woods N . Protein intake and incident frailty in the women’s health initiative observational study. J Am Geriatr Soc. (2010) 58:1063–71. doi: 10.1111/j.1532-5415.2010.02866.x, 20487071 PMC2924946

[17] Shikany JM Barrett-Connor E Ensrud KE Cawthon PM Lewis CE Dam T-TL . Macronutrients, diet quality, and frailty in older men. J Gerontol A Biol Sci Med Sci. (2013) 69:695–701. doi: 10.1093/gerona/glt196, 24304504 PMC4022097

[18] Chan R Leung J Woo J. Dietary patterns and risk of frailty in Chinese community-dwelling older people in Hong Kong: a prospective cohort study. Nutrients. (2015) 7:7070–84. doi: 10.3390/nu7085326, 26305253 PMC4555165

[19] Lorenzo-López L Maseda A de Labra C Regueiro-Folgueira L Rodríguez-Villamil JL Millán-Calenti JC. Nutritional determinants of frailty in older adults: a systematic review. BMC Geriatr. (2017) 17:108. doi: 10.1186/s12877-017-0496-2, 28506216 PMC5433026

[20] Ni Lochlainn M Cox NJ Wilson T Hayhoe RPG Ramsay SE Granic A . Nutrition and frailty: opportunities for prevention and treatment. Nutrients. (2021) 13:2349. doi: 10.3390/nu13072349, 34371858 PMC8308545

[21] García-Esquinas E Rahi B Peres K Colpo M Dartigues J-F Bandinelli S . Consumption of fruit and vegetables and risk of frailty: a dose-response analysis of 3 prospective cohorts of community-dwelling older adults. Am J Clin Nutr. (2016) 104:132–42. doi: 10.3945/ajcn.115.125781, 27194305

[22] Sandoval-Insausti H Blanco-Rojo R Graciani A López-García E Moreno-Franco B Laclaustra M . Ultra-processed food consumption and incident frailty: a prospective cohort study of older adults. J Gerontol A Biol Sci Med Sci. (2019) 75:1126–33. doi: 10.1093/gerona/glz140, 31132092

[23] Struijk EA Fung TT Rodríguez-Artalejo F Bischoff-Ferrari HA Hu FB Willett WC . Protein intake and risk of frailty among older women in the nurses’ health study. J Cachexia Sarcopenia Muscle. (2022) 13:1752–61. doi: 10.1002/jcsm.12972, 35318829 PMC9178161

[24] Reynolds A Mann J Cummings J Winter N Mete E Te Morenga L. Carbohydrate quality and human health: a series of systematic reviews and meta-analyses. Lancet. (2019) 393:434–45. doi: 10.1016/S0140-6736(18)31809-9, 30638909

[25] Ludwig DS. The glycemic index: physiological mechanisms relating to obesity, diabetes, and cardiovascular disease. JAMA. (2002) 287:2414. doi: 10.1001/jama.287.18.2414, 11988062

[26] Micha R Peñalvo JL Cudhea F Imamura F Rehm CD Mozaffarian D. Association between dietary factors and mortality from heart disease, stroke, and type 2 diabetes in the United States. JAMA. (2017) 317:912–24. doi: 10.1001/jama.2017.0947, 28267855 PMC5852674

[27] Volkert D Beck AM Cederholm T Cruz-Jentoft A Goisser S Hooper L . ESPEN guideline on clinical nutrition and hydration in geriatrics. Clin Nutr. (2019) 38:10–47. doi: 10.1016/j.clnu.2018.05.024, 30005900

[28] Bauer J Biolo G Cederholm T Cesari M Cruz-Jentoft AJ Morley JE . Evidence-based recommendations for optimal dietary protein intake in older people: a position paper from the PROT-AGE study group. J Am Med Dir Assoc. (2013) 14:542–59. doi: 10.1016/j.jamda.2013.05.021, 23867520

[29] Cruz-Jentoft AJ Bahat G Bauer J Boirie Y Bruyère O Cederholm T . Sarcopenia: revised european consensus on definition and diagnosis. Age Ageing. (2018) 48:16–31. doi: 10.1093/ageing/afy169, 30312372 PMC6322506

[30] Sonnega A Faul J Ofstedal M Langa K Phillips J Weir D. Cohort profile: the health and retirement study (HRS). Int J Epidemiol. (2014) 43:576–85. doi: 10.1093/ije/dyu067, 24671021 PMC3997380

[31] Fisher GG Ryan LH. Overview of the health and retirement study and introduction to the special issue. Work Aging Retire. (2017) 4:1–9. doi: 10.1093/workar/wax032, 29423243 PMC5798643

[32] Wang H Sun R Yan B Wei Y Wu Y Ling F . Digital exclusion and frailty among older adults: findings from four longitudinal cohort studies. Age Ageing. (2026) 55:afag133. doi: 10.1093/ageing/afag133, 42228905

[33] Jackson C. Multi-state models for panel data: the msm package for R. J Stat Softw. (2011) 38:1–29. doi: 10.18637/jss.v038.i08

[34] Putter H Fiocco M Geskus R. Tutorial in biostatistics: competing risks and multi-state models. Stat Med. (2006) 26:2389–430. doi: 10.1002/sim.2712, 17031868

[35] Andersen PK Keiding N. Multi-state models for event history analysis. Stat Methods Med Res. (2002) 11:91–115. doi: 10.1191/0962280202SM276ra 12040698

[36] Meira-Machado L de Uña-Alvarez J Cadarso-Suárez C Andersen PK. Multi-state models for the analysis of time-to-event data. Stat Methods Med Res. (2008) 18:195–222. doi: 10.1177/0962280208092301, 18562394 PMC2692556

[37] Coelho-Júnior HJ Álvarez-Bustos A Rodríguez-Sánchez I Landi F Marzetti E. Muscle failure and risk of adverse outcomes in older adults: a derivation and multicohort validation study. Lancet Healthy Longev. (2026) 7:100843. doi: 10.1016/j.lanhl.2026.100843, 42119578

