# Supplementary material for: Protein density, carbohydrate quality, and frailty-state transitions among adults aged 50 years or older: a continuous-time multistate analysis of the health and retirement study
Source: Front Nutr. 2026 Sep 24;13:1926576. doi: 10.3389/fnut.2026.1926576 (PMC13645535; doi:10.3389/fnut.2026.1926576)
Supplement: Supplementary file 1 [file Data_Sheet_1.PDF]

Supplementary Information

Protein density, carbohydrate quality, and frailty-state transitions among adults aged 50 years or older: a continuous-time multistate analysis of the Health and Retirement Study

Supplementary Methods

The dietary profile score combined five energy-adjusted components from the 2013 nutrition assessment. Component z scores were calculated across all 8,035 nutrition records and combined as an equally weighted, directionally coded mean. All five components were complete. The composite was standardized using the mean and SD among 7,061 participants eligible for core-adjusted analyses; the SD was 0.616 score units. Quartiles and prediction percentiles were calculated across the 6,982 participants in the primary sample. Component-specific SDs were also calculated in the primary sample, with each participant contributing one baseline record.

The 28-item frailty index followed published deficit-accumulation methods. At each wave, the observed deficit sum was divided by the number of observed items. The index was calculated with at least 22 of 28 items and set to missing otherwise. Living states were robust for FI<0.10, pre-frail for 0.10<=FI<0.25, and frail for FI>=0.25.

Eligibility required baseline age of at least 50 years and a valid wave-12 living state. Follow-up was measured from each participant's wave-12 interview using RAND monthly midpoint dates for interviews. Deaths were modeled as exact events using the recorded RAND death dates.

The primary model allowed baseline transition intensities to vary across 0 to <2, 2 to <4, and at least 4 years of follow-up. Dietary associations were estimated separately for each transition and assumed constant across periods. Covariates comprised age, sex, education, baseline BMI, race, Hispanic ethnicity, current smoking, alcohol use, and moderate and light physical activity. Each covariate had a separate coefficient for each allowed transition. Age, education, and BMI were standardized continuous variables. Physical-activity frequencies (RAND r12mdactx and r12ltactx) were coded from 1 (every day) to 5 (never). Intermediate categories were more than once weekly, once weekly, and one to three times monthly. Standardized ordinal activity terms assumed a linear association with each log-transition intensity. Race was represented by indicator variables, with White participants as the reference group. Sex, Hispanic ethnicity, current smoking, and alcohol use were binary indicators. Alcohol use (r12drink) did not measure consumption quantity. Models were conditional on the initial frailty state and did not additionally adjust for continuous baseline FI. Core-adjusted models included age, sex, education, and baseline BMI. Core-adjusted and time-homogeneous models used the same complete-case sample as the primary model. Further sensitivity analyses required at least 25 of 28 observed FI items, omitted BMI, or applied delayed entry from year 2. For delayed entry, follow-up began at the first living-state assessment at or after year 2, with at least one subsequent state observation required. This analysis was conditional on survival and observation to the later entry time. Component analyses included each component separately or all five jointly, with the primary model's time periods and full adjustment. HRs were expressed per 1-SD higher intake density in the primary sample, without reversing added sugar or glycemic load. Benjamini-Hochberg correction was applied to 35 component-transition tests separately within each exploratory family: the separate component models and the mutually adjusted model.

Supplementary Table S1. Construction and interpretation of the study-specific protein-density and carbohydrate-quality score.

Component	Energy-adjusted measure	Contribution	Construct represented
Protein	Protein density per 1,000 kcal	+z	Protein density
Fiber	Fiber density per 1,000 kcal	+z	Carbohydrate quality
Whole grains	Whole-grain density per 1,000 kcal	+z	Carbohydrate quality
Added sugar	Added-sugar density per 1,000 kcal	-z	Lower added-sugar burden
Glycemic load	Glycemic-load density per 1,000 kcal	-z	Lower glycemic burden

Score = [z(protein density) + z(fiber density) + z(whole-grain density) - z(added-sugar density) - z(glycemic-load density)] / 5. Component z scores were calculated across all 8,035 nutrition records. Composite-score HRs use the SD of 0.616 score units among 7,061 participants eligible for core-adjusted analyses. Component-specific HRs describe higher intake density; added sugar and glycemic load were not reverse-coded in those analyses.

Supplementary Table S2. Operational coding for the 28 frailty-index deficit items.

No.	Deficit item	Domain	Variable stem	Coding
1	hypertension	chronic disease	hibp	Yes=1, No=0
2	diabetes	chronic disease	diab	Yes=1, No=0
3	cancer	chronic disease	cancr	Yes=1, No=0
4	arthritis	chronic disease	arthr	Yes=1, No=0
5	chronic lung disease	chronic disease	lung	Yes=1, No=0
6	psychiatric problem	chronic disease	psych	Yes=1, No=0
7	memory related disease	chronic disease	alzhe/demen	Alzheimer's disease or dementia reported=1; neither=0
8	vision problem	sensory	sight	Blind, poor, or fair=1; good, very good, or excellent=0
9	hearing problem	sensory	hearing	Deaf, poor, or fair=1; good, very good, or excellent=0
10	poor general health	general health	shlt	Poor=1; fair, good, very good, or excellent=0
11	difficulty dressing	badl	dressa	Any difficulty=1; no difficulty=0
12	difficulty bathing	badl	batha	Any difficulty=1; no difficulty=0
13	difficulty eating	badl	eata	Any difficulty=1; no difficulty=0
14	difficulty getting in out bed	badl	beda	Any difficulty=1; no difficulty=0
15	difficulty using toilet	badl	toilta	Any difficulty=1; no difficulty=0
16	difficulty managing money	iadl	moneya	Any difficulty=1; no difficulty=0
17	difficulty taking medications	iadl	medsa	Any difficulty=1; no difficulty=0
18	difficulty shopping groceries	iadl	shopa	Any difficulty=1; no difficulty=0
19	difficulty preparing meals	iadl	mealsa	Any difficulty=1; no difficulty=0
20	difficulty walking one block	mobility	walk1a	Any difficulty=1; no difficulty=0
21	difficulty getting up chair	mobility	chaira	Any difficulty=1; no difficulty=0
22	difficulty climbing several flights	mobility	climsa	Any difficulty=1; no difficulty=0
23	difficulty lifting 10 pounds	mobility	lifta	Any difficulty=1; no difficulty=0
24	difficulty stooping	mobility	stoopa	Any difficulty=1; no difficulty=0
25	difficulty reaching arms	mobility	armsa	Any difficulty=1; no difficulty=0
26	difficulty picking dime	mobility	dimea	Any difficulty=1; no difficulty=0
27	depression	depression	cesd	CESD-8 >=4=1; <4=0
28	cognition	cognition	imrc/dlrc/date naming	(24 - (immediate recall + delayed recall + 4 date naming items)) / 24

Most deficits were binary. Cognition was coded continuously from 0 to 1. FI, frailty index; ADL, activity of daily living; IADL, instrumental activity of daily living.

Supplementary Table S3. Frailty-index availability and distributions among participants aged 50 years or older.

Wave (nominal year)	Age-eligible n	FI available n (%)	Mean observed items	FI mean (SD)	Robust n (%)	Pre-frail n (%)	Frail n (%)	Interview-date range
12 (2014)	7,434	7,431 (100.0%)	27.35	0.201 (0.163)	2,346 (31.6%)	2,840 (38.2%)	2,245 (30.2%)	2014-03-15 to 2015-04-15
13 (2016)	7,434	6,518 (87.7%)	27.37	0.207 (0.165)	1,945 (29.8%)	2,539 (39.0%)	2,034 (31.2%)	2016-03-15 to 2017-11-15
14 (2018)	7,434	5,519 (74.2%)	27.42	0.212 (0.165)	1,573 (28.5%)	2,143 (38.8%)	1,803 (32.7%)	2018-04-15 to 2019-06-15
15 (2020)	7,434	5,041 (67.8%)	27.36	0.207 (0.164)	1,476 (29.3%)	1,991 (39.5%)	1,574 (31.2%)	2020-03-15 to 2021-05-15

Availability counts refer to the 7,434 age-eligible participants before complete-case selection; the primary model included 6,982 participants. State percentages use participants with an available frailty index at each wave. Interview dates are RAND monthly midpoint dates.

Supplementary Table S4. Participant selection and ascertainment of interview and death dates.

Panel	Metric	Value	Definition / exclusions
A. Cohort	Linked participants aged >=50 years at baseline	7,434	
A. Cohort	Valid wave-12 (2014) frailty state	7,431	Excluded 3
A. Cohort	Complete core covariates and dietary score	7,307	Excluded 124
A. Cohort	At least two valid state observations; eligible for core-adjusted analyses	7,061	Excluded 246
A. Cohort	Complete covariates for full adjustment; primary sample	6,982	Excluded a further 79 participants
B. Timing	Wave-12 interview range	2014-03-15 to 2015-04-15	RAND monthly midpoint dates
B. Timing	Wave-15 interview range	2020-03-15 to 2021-05-15	RAND monthly midpoint dates
B. Timing	Recorded death dates available	1,876	RAND raddate
B. Timing	Exit-interview year later than recorded death year	1,043	Among records with both fields
B. Timing	Median difference between exit-interview year and death year	1 year	Death times based on RAND death dates
B. Timing	Administrative end date	2021-05-15	Final observed interview month

The primary sample required a valid wave-12 living state, at least two valid state observations, and complete covariates for full adjustment. Date summaries refer to the 7,434 age-eligible participants before complete-case selection. The count of available death dates is not restricted to deaths during analytic follow-up. Death times were based on recorded RAND death dates, not exit-interview years.

Supplementary Table S5. Model convergence and model-specific sample sizes.

Model	Participants	Observations	Deaths	Optimizer code	Hessian and gradient diagnostics
Fully adjusted, time-homogeneous	6982	25108	1487	0	Positive definite; maximum absolute gradient 0.0018
Fully adjusted, piecewise (primary)	6982	25108	1487	0	Positive definite; maximum absolute gradient 0.0046
Core-adjusted, time-homogeneous	6982	25108	1487	0	Positive definite; maximum absolute gradient 0.0019
Core-adjusted, piecewise	6982	25108	1487	0	Positive definite; maximum absolute gradient 0.0012
Fully adjusted, piecewise, without score	6982	25108	1487	0	Positive definite; maximum absolute gradient 0.0009
Fully adjusted, piecewise, score quartiles	6982	25108	1487	0	Positive definite; maximum absolute gradient 0.0008
Added sugar	6982	25108	1487	0	Positive definite; maximum absolute gradient 0.0017
Fiber	6982	25108	1487	0	Positive definite; maximum absolute gradient 0.0026
Glycemic load	6982	25108	1487	0	Positive definite; maximum absolute gradient 0.0009
Protein	6982	25108	1487	0	Positive definite; maximum absolute gradient 0.0011
Whole grains	6982	25108	1487	0	Positive definite; maximum absolute gradient 0.0010
Mutually adjusted components	6982	25108	1487	0	Positive definite; maximum absolute gradient 0.0010
Delayed entry from year 2	5920	16211	932	0	Positive definite; maximum absolute gradient 0.0009
BMI omitted	6982	25108	1487	0	Positive definite; maximum absolute gradient 0.0010
At least 25 observed FI items	6942	24889	1462	0	Positive definite; maximum absolute gradient 0.0009

All models converged (optimizer code 0), with a positive-definite Hessian for the free parameters, finite covariance estimates, and maximum absolute likelihood gradients below 0.1. Covariate coefficients were estimated separately for each transition. Reported observation counts exclude auxiliary records introduced at period boundaries for likelihood calculation in msm.

Supplementary Table S6. Complete baseline characteristics by dietary profile score quartile.

Characteristic	Overall	Q1	Q2	Q3	Q4	P
Participants	6982	1746	1745	1745	1746	
Dietary profile score	0.00 (0.62)	-0.80 (0.42)	-0.14 (0.11)	0.22 (0.10)	0.73 (0.28)	<0.001
Age, years	69.00 (10.49)	68.89 (10.84)	69.83 (10.61)	69.40 (10.30)	67.89 (10.10)	<0.001
Female	4103 (58.8%)	937 (53.7%)	1010 (57.9%)	1060 (60.7%)	1096 (62.8%)	<0.001
Hispanic ethnicity	818 (11.7%)	144 (8.2%)	164 (9.4%)	205 (11.7%)	305 (17.5%)	<0.001
Race: White	5275 (75.6%)	1170 (67.0%)	1297 (74.3%)	1408 (80.7%)	1400 (80.2%)	<0.001
Race: Black	1193 (17.1%)	449 (25.7%)	333 (19.1%)	220 (12.6%)	191 (10.9%)	
Race: Other	514 (7.4%)	127 (7.3%)	115 (6.6%)	117 (6.7%)	155 (8.9%)	
Education, years	12.89 (3.03)	12.30 (2.78)	12.83 (2.88)	13.26 (2.91)	13.19 (3.42)	<0.001
BMI, kg/m2	28.60 (6.08)	28.44 (6.30)	28.58 (5.96)	28.76 (6.01)	28.60 (6.05)	0.493
Current smoking	771 (11.0%)	324 (18.6%)	209 (12.0%)	127 (7.3%)	111 (6.4%)	<0.001
Alcohol use	3792 (54.3%)	821 (47.0%)	936 (53.6%)	1022 (58.6%)	1013 (58.0%)	<0.001
Moderate activity: Every day	503 (7.2%)	98 (5.6%)	119 (6.8%)	150 (8.6%)	136 (7.8%)	<0.001
Moderate activity: More than once a week	2833 (40.6%)	536 (30.7%)	660 (37.8%)	774 (44.4%)	863 (49.4%)	
Moderate activity: Once a week	1243 (17.8%)	315 (18.0%)	313 (17.9%)	298 (17.1%)	317 (18.2%)	
Moderate activity: One to three times a month	842 (12.1%)	254 (14.5%)	228 (13.1%)	206 (11.8%)	154 (8.8%)	
Moderate activity: Never	1561 (22.4%)	543 (31.1%)	425 (24.4%)	317 (18.2%)	276 (15.8%)	
Light activity: Every day	569 (8.1%)	107 (6.1%)	146 (8.4%)	155 (8.9%)	161 (9.2%)	<0.001
Light activity: More than once a week	3121 (44.7%)	674 (38.6%)	720 (41.3%)	829 (47.5%)	898 (51.4%)	
Light activity: Once a week	1919 (27.5%)	532 (30.5%)	495 (28.4%)	465 (26.6%)	427 (24.5%)	
Light activity: One to three times a month	573 (8.2%)	163 (9.3%)	165 (9.5%)	137 (7.9%)	108 (6.2%)	
Light activity: Never	800 (11.5%)	270 (15.5%)	219 (12.6%)	159 (9.1%)	152 (8.7%)	
Frailty index	0.20 (0.16)	0.23 (0.18)	0.21 (0.16)	0.19 (0.15)	0.17 (0.15)	<0.001
Observed FI items	27.36 (0.73)	27.29 (0.77)	27.38 (0.77)	27.40 (0.71)	27.38 (0.66)	<0.001
Robust	2196 (31.5%)	460 (26.3%)	506 (29.0%)	566 (32.4%)	664 (38.0%)	<0.001
Pre-frail	2664 (38.2%)	624 (35.7%)	675 (38.7%)	693 (39.7%)	672 (38.5%)	
Frail	2122 (30.4%)	662 (37.9%)	564 (32.3%)	486 (27.9%)	410 (23.5%)	
Death during follow-up	1487 (21.3%)	472 (27.0%)	411 (23.6%)	349 (20.0%)	255 (14.6%)	<0.001

Values are mean (SD) or n (%). Quartiles were defined across participants in the primary sample. P values were calculated using one-way ANOVA for continuous variables and chi-square tests for categorical variables. All covariates included in the primary model were complete in this sample.

Supplementary Table S7. Observed state changes between successive assessments in the primary sample.

From state	To state	n	Row %	Model representation
robust	robust	4,002	73.7	
robust	pre-frail	1,213	22.3	
robust	frail	90	1.7	Latent intermediate state
robust	death	126	2.3	
pre-frail	robust	858	12.3	
pre-frail	pre-frail	4,473	63.9	
pre-frail	frail	1,302	18.6	
pre-frail	death	371	5.3	
frail	robust	20	0.4	Latent intermediate state
frail	pre-frail	806	14.2	
frail	frail	3,875	68.1	
frail	death	990	17.4	

Non-adjacent living-state observations were represented in the continuous-time model as passage through the intermediate state during the observation interval.

Supplementary Table S8. Model fit comparisons and likelihood-ratio tests.

Panel	Model / comparison	Participants	Observations	Log likelihood	df	AIC	LR	P
Model fit	Fully adjusted, time-homogeneous	6982	25108	-15953.78	91	32089.56		
Model fit	Fully adjusted, piecewise (primary)	6982	25108	-15776.77	105	31763.53		
Model fit	Core-adjusted, time-homogeneous	6982	25108	-16194.53	42	32473.05		
Model fit	Core-adjusted, piecewise	6982	25108	-16039.49	56	32190.99		
Model fit	Fully adjusted, piecewise, without score	6982	25108	-15788.81	98	31773.62		
Model fit	Fully adjusted, piecewise, score quartiles	6982	25108	-15772.65	119	31783.30		
Model fit	Added sugar	6982	25108	-15778.82	105	31767.63		
Model fit	Fiber	6982	25108	-15766.20	105	31742.40		
Model fit	Glycemic load	6982	25108	-15783.02	105	31776.05		
Model fit	Protein	6982	25108	-15783.25	105	31776.49		
Model fit	Whole grains	6982	25108	-15784.15	105	31778.29		
Model fit	Mutually adjusted components	6982	25108	-15740.34	133	31746.67		
Model fit	Delayed entry from year 2	5920	16211	-9020.79	98	18237.58		
Model fit	BMI omitted	6982	25108	-15882.50	98	31960.99		
Model fit	At least 25 observed FI items	6942	24889	-15588.03	105	31386.06		
LRT	Piecewise vs time-homogeneous, full adjustment	6,982	25,108		14		354.02	<0.001
LRT	Piecewise vs time-homogeneous, core adjustment	6,982	25,108		14		310.07	<0.001
LRT	Full vs core adjustment, piecewise models	6,982	25,108		49		525.46	<0.001
LRT	Continuous score vs score omitted	6,982	25,108		7		24.08	0.001
LRT	Score quartiles vs score omitted	6,982	25,108		21		32.31	0.054
LRT	Five components vs components omitted	6,982	25,108		35		96.94	<0.001

Likelihood-ratio tests compared models fitted to identical participants and observed records. Auxiliary records introduced at period boundaries do not represent additional observations. AIC values were compared only for models using identical participants and observations. Models with different eligibility or follow-up criteria are not directly comparable.

Supplementary Table S9. Transition-specific dietary profile score hazard ratios across model specifications.

Model	Exposure term	Transition	HR (95% CI)	P
Fully adjusted, piecewise (primary)	Score per SD	Robust to pre-frail	0.933 (0.875-0.995)	0.034
Fully adjusted, piecewise (primary)	Score per SD	Robust to death	0.848 (0.625-1.150)	0.288
Fully adjusted, piecewise (primary)	Score per SD	Pre-frail to robust	1.024 (0.950-1.105)	0.532
Fully adjusted, piecewise (primary)	Score per SD	Pre-frail to frail	0.978 (0.922-1.038)	0.464
Fully adjusted, piecewise (primary)	Score per SD	Pre-frail to death	0.809 (0.662-0.989)	0.039
Fully adjusted, piecewise (primary)	Score per SD	Frail to pre-frail	1.050 (0.979-1.127)	0.174
Fully adjusted, piecewise (primary)	Score per SD	Frail to death	0.939 (0.880-1.002)	0.057
Fully adjusted, time-homogeneous	Score per SD	Robust to pre-frail	0.934 (0.876-0.996)	0.038
Fully adjusted, time-homogeneous	Score per SD	Robust to death	0.856 (0.617-1.187)	0.350
Fully adjusted, time-homogeneous	Score per SD	Pre-frail to robust	1.025 (0.950-1.105)	0.529
Fully adjusted, time-homogeneous	Score per SD	Pre-frail to frail	0.976 (0.920-1.035)	0.422
Fully adjusted, time-homogeneous	Score per SD	Pre-frail to death	0.831 (0.670-1.030)	0.090
Fully adjusted, time-homogeneous	Score per SD	Frail to pre-frail	1.049 (0.978-1.126)	0.179
Fully adjusted, time-homogeneous	Score per SD	Frail to death	0.944 (0.885-1.008)	0.084
Core-adjusted, time-homogeneous	Score per SD	Robust to pre-frail	0.908 (0.853-0.966)	0.002
Core-adjusted, time-homogeneous	Score per SD	Robust to death	0.704 (0.515-0.960)	0.027
Core-adjusted, time-homogeneous	Score per SD	Pre-frail to robust	1.060 (0.986-1.140)	0.117
Core-adjusted, time-homogeneous	Score per SD	Pre-frail to frail	0.917 (0.866-0.970)	0.003
Core-adjusted, time-homogeneous	Score per SD	Pre-frail to death	0.841 (0.672-1.054)	0.133
Core-adjusted, time-homogeneous	Score per SD	Frail to pre-frail	1.110 (1.039-1.187)	0.002
Core-adjusted, time-homogeneous	Score per SD	Frail to death	0.891 (0.837-0.949)	<0.001
Core-adjusted, piecewise	Score per SD	Robust to pre-frail	0.908 (0.853-0.966)	0.002
Core-adjusted, piecewise	Score per SD	Robust to death	0.686 (0.510-0.922)	0.013
Core-adjusted, piecewise	Score per SD	Pre-frail to robust	1.059 (0.985-1.139)	0.121
Core-adjusted, piecewise	Score per SD	Pre-frail to frail	0.916 (0.866-0.970)	0.003
Core-adjusted, piecewise	Score per SD	Pre-frail to death	0.829 (0.658-1.044)	0.110
Core-adjusted, piecewise	Score per SD	Frail to pre-frail	1.111 (1.039-1.188)	0.002
Core-adjusted, piecewise	Score per SD	Frail to death	0.879 (0.825-0.936)	<0.001
Fully adjusted, piecewise, score quartiles	Q2 vs Q1	Robust to pre-frail	0.922 (0.778-1.093)	0.351
Fully adjusted, piecewise, score quartiles	Q2 vs Q1	Robust to death	0.800 (0.354-1.806)	0.591
Fully adjusted, piecewise, score quartiles	Q2 vs Q1	Pre-frail to robust	1.074 (0.872-1.323)	0.504
Fully adjusted, piecewise, score quartiles	Q2 vs Q1	Pre-frail to frail	0.959 (0.819-1.123)	0.602
Fully adjusted, piecewise, score quartiles	Q2 vs Q1	Pre-frail to death	0.822 (0.474-1.424)	0.483
Fully adjusted, piecewise, score quartiles	Q2 vs Q1	Frail to pre-frail	1.125 (0.920-1.375)	0.251
Fully adjusted, piecewise, score quartiles	Q2 vs Q1	Frail to death	1.040 (0.882-1.227)	0.639
Fully adjusted, piecewise, score quartiles	Q3 vs Q1	Robust to pre-frail	0.835 (0.704-0.991)	0.039
Fully adjusted, piecewise, score quartiles	Q3 vs Q1	Robust to death	0.849 (0.377-1.913)	0.693
Fully adjusted, piecewise, score quartiles	Q3 vs Q1	Pre-frail to robust	1.031 (0.836-1.272)	0.774
Fully adjusted, piecewise, score quartiles	Q3 vs Q1	Pre-frail to frail	1.084 (0.924-1.272)	0.323
Fully adjusted, piecewise, score quartiles	Q3 vs Q1	Pre-frail to death	0.830 (0.483-1.428)	0.501
Fully adjusted, piecewise, score quartiles	Q3 vs Q1	Frail to pre-frail	1.121 (0.911-1.379)	0.281
Fully adjusted, piecewise, score quartiles	Q3 vs Q1	Frail to death	0.924 (0.774-1.104)	0.385
Fully adjusted, piecewise, score quartiles	Q4 vs Q1	Robust to pre-frail	0.807 (0.680-0.958)	0.014
Fully adjusted, piecewise, score quartiles	Q4 vs Q1	Robust to death	0.527 (0.213-1.303)	0.166
Fully adjusted, piecewise, score quartiles	Q4 vs Q1	Pre-frail to robust	1.085 (0.883-1.335)	0.437
Fully adjusted, piecewise, score quartiles	Q4 vs Q1	Pre-frail to frail	0.905 (0.766-1.070)	0.244
Fully adjusted, piecewise, score quartiles	Q4 vs Q1	Pre-frail to death	0.693 (0.401-1.197)	0.189
Fully adjusted, piecewise, score quartiles	Q4 vs Q1	Frail to pre-frail	1.142 (0.926-1.408)	0.214
Fully adjusted, piecewise, score quartiles	Q4 vs Q1	Frail to death	0.844 (0.693-1.028)	0.091
Delayed entry from year 2	Score per SD	Robust to pre-frail	0.940 (0.859-1.028)	0.173
Delayed entry from year 2	Score per SD	Robust to death	0.849 (0.563-1.281)	0.435
Delayed entry from year 2	Score per SD	Pre-frail to robust	1.039 (0.940-1.148)	0.455
Delayed entry from year 2	Score per SD	Pre-frail to frail	1.024 (0.945-1.110)	0.559
Delayed entry from year 2	Score per SD	Pre-frail to death	0.787 (0.602-1.029)	0.080
Delayed entry from year 2	Score per SD	Frail to pre-frail	1.059 (0.964-1.164)	0.234
Delayed entry from year 2	Score per SD	Frail to death	0.939 (0.867-1.018)	0.128
At least 25 observed FI items	Score per SD	Robust to pre-frail	0.935 (0.876-0.997)	0.040
At least 25 observed FI items	Score per SD	Robust to death	0.847 (0.629-1.141)	0.275
At least 25 observed FI items	Score per SD	Pre-frail to robust	1.030 (0.954-1.111)	0.449
At least 25 observed FI items	Score per SD	Pre-frail to frail	0.985 (0.928-1.045)	0.621
At least 25 observed FI items	Score per SD	Pre-frail to death	0.798 (0.654-0.973)	0.025
At least 25 observed FI items	Score per SD	Frail to pre-frail	1.055 (0.983-1.132)	0.141
At least 25 observed FI items	Score per SD	Frail to death	0.942 (0.882-1.005)	0.072
Fully adjusted, piecewise, BMI omitted	Score per SD	Robust to pre-frail	0.939 (0.880-1.000)	0.051
Fully adjusted, piecewise, BMI omitted	Score per SD	Robust to death	0.847 (0.616-1.164)	0.306
Fully adjusted, piecewise, BMI omitted	Score per SD	Pre-frail to robust	1.009 (0.935-1.088)	0.825
Fully adjusted, piecewise, BMI omitted	Score per SD	Pre-frail to frail	0.980 (0.924-1.040)	0.510

Model	Exposure term	Transition	HR (95% CI)	P
Fully adjusted, piecewise, BMI omitted	Score per SD	Pre-frail to death	0.827 (0.676-1.012)	0.065
Fully adjusted, piecewise, BMI omitted	Score per SD	Frail to pre-frail	1.039 (0.968-1.114)	0.291
Fully adjusted, piecewise, BMI omitted	Score per SD	Frail to death	0.920 (0.862-0.982)	0.012

All covariate coefficients are transition-specific. Composite-score HRs use an SD of 0.616 score units among participants eligible for core-adjusted analyses. Q1 is the reference category for quartile estimates. Delayed entry begins at the first observed living-state assessment at or after year 2. Estimates are conditional on survival and observation to that time and do not represent all baseline participants.

Supplementary Table S10. Component-specific associations in fully adjusted piecewise models.

Component per SD	Transition	HR (95% CI)	P	BH-adjusted P
Protein	Robust to pre-frail	1.005 (0.943-1.072)	0.867	0.928
Protein	Robust to death	0.939 (0.676-1.304)	0.706	0.824
Protein	Pre-frail to robust	0.978 (0.911-1.049)	0.533	0.777
Protein	Pre-frail to frail	1.012 (0.957-1.069)	0.684	0.824
Protein	Pre-frail to death	0.749 (0.591-0.951)	0.018	0.205
Protein	Frail to pre-frail	0.949 (0.885-1.016)	0.133	0.389
Protein	Frail to death	0.998 (0.940-1.060)	0.945	0.945
Fiber	Robust to pre-frail	0.882 (0.830-0.937)	<0.001	0.002
Fiber	Robust to death	0.915 (0.661-1.266)	0.591	0.796
Fiber	Pre-frail to robust	1.067 (0.990-1.150)	0.088	0.389
Fiber	Pre-frail to frail	0.948 (0.887-1.012)	0.108	0.389
Fiber	Pre-frail to death	0.871 (0.665-1.141)	0.316	0.546
Fiber	Frail to pre-frail	1.082 (1.005-1.165)	0.037	0.318
Fiber	Frail to death	0.939 (0.872-1.010)	0.092	0.389
Whole grains	Robust to pre-frail	0.959 (0.903-1.019)	0.173	0.403
Whole grains	Robust to death	1.028 (0.777-1.360)	0.844	0.928
Whole grains	Pre-frail to robust	0.964 (0.897-1.037)	0.327	0.546
Whole grains	Pre-frail to frail	0.996 (0.940-1.056)	0.896	0.928
Whole grains	Pre-frail to death	0.860 (0.681-1.087)	0.206	0.452
Whole grains	Frail to pre-frail	1.053 (0.985-1.127)	0.129	0.389
Whole grains	Frail to death	0.966 (0.902-1.034)	0.315	0.546
Added sugar	Robust to pre-frail	1.097 (1.028-1.170)	0.005	0.089
Added sugar	Robust to death	1.187 (0.892-1.580)	0.241	0.496
Added sugar	Pre-frail to robust	0.973 (0.900-1.052)	0.488	0.742
Added sugar	Pre-frail to frail	1.004 (0.948-1.063)	0.901	0.928
Added sugar	Pre-frail to death	1.052 (0.847-1.306)	0.648	0.810
Added sugar	Frail to pre-frail	0.934 (0.870-1.003)	0.062	0.362
Added sugar	Frail to death	1.045 (0.984-1.111)	0.151	0.397
Glycemic load	Robust to pre-frail	0.939 (0.883-0.999)	0.045	0.318
Glycemic load	Robust to death	1.175 (0.888-1.555)	0.260	0.505
Glycemic load	Pre-frail to robust	0.949 (0.882-1.021)	0.159	0.397
Glycemic load	Pre-frail to frail	1.024 (0.965-1.086)	0.440	0.700
Glycemic load	Pre-frail to death	1.061 (0.857-1.314)	0.587	0.796
Glycemic load	Frail to pre-frail	0.982 (0.912-1.057)	0.623	0.807
Glycemic load	Frail to death	1.055 (0.988-1.127)	0.113	0.389

HRs are expressed per SD higher intake density, including for added sugar and glycemic load. Each component was modeled separately, with the same covariates and time periods as the primary model. Benjamini-Hochberg (BH) correction was applied across the 35 component-transition tests in this exploratory family.

Supplementary Table S11. Mutually adjusted component associations in the fully adjusted piecewise model.

Component per SD	Transition	HR (95% CI)	P	BH-adjusted P
Protein	Robust to pre-frail	0.994 (0.924-1.069)	0.865	0.865
Protein	Robust to death	1.040 (0.710-1.523)	0.840	0.865
Protein	Pre-frail to robust	0.942 (0.867-1.023)	0.155	0.339
Protein	Pre-frail to frail	1.027 (0.962-1.096)	0.425	0.629
Protein	Pre-frail to death	0.735 (0.562-0.960)	0.024	0.105
Protein	Frail to pre-frail	0.880 (0.808-0.959)	0.003	0.105
Protein	Frail to death	1.047 (0.975-1.125)	0.202	0.404
Fiber	Robust to pre-frail	0.911 (0.842-0.986)	0.021	0.105
Fiber	Robust to death	0.863 (0.581-1.283)	0.467	0.629
Fiber	Pre-frail to robust	1.129 (1.030-1.238)	0.010	0.105
Fiber	Pre-frail to frail	0.908 (0.836-0.986)	0.022	0.105
Fiber	Pre-frail to death	0.908 (0.681-1.209)	0.509	0.659
Fiber	Frail to pre-frail	1.074 (0.976-1.182)	0.143	0.339
Fiber	Frail to death	0.916 (0.835-1.004)	0.062	0.217
Whole grains	Robust to pre-frail	1.055 (0.981-1.134)	0.147	0.339
Whole grains	Robust to death	1.081 (0.782-1.496)	0.637	0.796
Whole grains	Pre-frail to robust	0.939 (0.861-1.024)	0.154	0.339
Whole grains	Pre-frail to frail	1.009 (0.942-1.082)	0.792	0.865
Whole grains	Pre-frail to death	0.895 (0.684-1.170)	0.416	0.629
Whole grains	Frail to pre-frail	1.035 (0.956-1.121)	0.391	0.629
Whole grains	Frail to death	0.970 (0.894-1.052)	0.459	0.629
Added sugar	Robust to pre-frail	1.110 (1.016-1.213)	0.022	0.105
Added sugar	Robust to death	1.061 (0.714-1.578)	0.769	0.865
Added sugar	Pre-frail to robust	1.022 (0.920-1.134)	0.688	0.812
Added sugar	Pre-frail to frail	0.952 (0.882-1.028)	0.208	0.404
Added sugar	Pre-frail to death	0.854 (0.652-1.120)	0.254	0.468
Added sugar	Frail to pre-frail	0.953 (0.864-1.052)	0.341	0.596
Added sugar	Frail to death	0.984 (0.908-1.066)	0.696	0.812
Glycemic load	Robust to pre-frail	0.904 (0.832-0.983)	0.019	0.105
Glycemic load	Robust to death	1.158 (0.792-1.692)	0.450	0.629
Glycemic load	Pre-frail to robust	0.907 (0.822-1.002)	0.054	0.211
Glycemic load	Pre-frail to frail	1.075 (0.991-1.165)	0.081	0.258
Glycemic load	Pre-frail to death	1.022 (0.803-1.301)	0.859	0.865
Glycemic load	Frail to pre-frail	0.916 (0.824-1.019)	0.106	0.309
Glycemic load	Frail to death	1.117 (1.021-1.223)	0.016	0.105

HRs are expressed per SD higher intake density, including for added sugar and glycemic load. All five components were included jointly, with the same covariates and time periods as the primary model. Benjamini-Hochberg (BH) correction was applied across the 35 component-transition tests in this exploratory family.

Supplementary Table S12. Estimated annual transition intensities by follow-up period at a reference covariate profile.

Transition	0 to <2 years	2 to <4 years	>=4 years
Robust to pre-frail	0.1950	0.2216	0.2221
Robust to death	0.0021	0.0070	0.0106
Pre-frail to robust	0.0795	0.0751	0.0796
Pre-frail to frail	0.1192	0.1407	0.1385
Pre-frail to death	0.0057	0.0071	0.0208
Frail to pre-frail	0.1347	0.1165	0.1183
Frail to death	0.0323	0.0452	0.0925

The dietary profile score is set to zero on its standardized scale, and other covariates are fixed at their participant-level means in the primary sample. Rates describe this reference profile and are not marginal population incidence rates.

Supplementary Table S13. Pearson correlations and component-level variance inflation factors in 6,982 participants.

Component	Protein	Fiber	Whole grains	Added sugar	Glycemic load	VIF
Protein	1.000	0.064	0.059	-0.375	-0.488	1.38
Fiber	0.064	1.000	0.507	-0.430	0.153	1.72
Whole grains	0.059	0.507	1.000	-0.271	0.197	1.44
Added sugar	-0.375	-0.430	-0.271	1.000	0.467	2.00
Glycemic load	-0.488	0.153	0.197	0.467	1.000	1.97

Variance inflation factors (VIFs) quantify collinearity among the five component densities. Each component was regressed on the remaining four components to calculate its VIF.
